# Recurrent Takotsubo Syndrome and Pulmonary Embolism Revealing a Pheochromocytoma: A Diagnostic Pitfall

**DOI:** 10.7759/cureus.108731

**Published:** 2026-05-12

**Authors:** Alfakihi Ahmed Said IsmaIl, Jonathan Moyambi, Salim Arous, Abdenasser Drighil, Brahim Meftout

**Affiliations:** 1 Cardiology, CHU Ibn Rochd, Casablanca, MAR; 2 Cardiovascular Disease, CHU Ibn Rochd, Casablanca, MAR; 3 Cardiology, Centre Hospitalier Sud Francilien, Corbeil-Essonnes, FRA

**Keywords:** cardiogenic shock, left ventricular dysfunction, multimodal imaging, pheochromocytoma, pulmonary embolism, stress cardiomyopathy

## Abstract

Stress cardiomyopathy (Takotsubo syndrome or TTS) is an acute and reversible left ventricular dysfunction typically triggered by emotional or physical stress. Although most cases are idiopathic, secondary causes should be suspected in cases of recurrence, unusual severity, or atypical presentation. The association between TTS and pulmonary embolism (PE) is rare and may lead to diagnostic pitfalls, particularly when left ventricular dysfunction is misinterpreted as a marker of embolic severity. We report the case of a 46-year-old woman with two episodes of TTS one year apart. The second episode, presenting as a reverse Takotsubo pattern, was complicated by cardiogenic shock and multiorgan failure. A thoraco-abdominopelvic CT scan performed during etiological workup revealed a subsegmental PE without right ventricular dysfunction. The diagnosis of TTS was confirmed by ventriculography, demonstrating characteristic wall motion abnormalities in the absence of obstructive coronary artery disease. The diagnosis of pheochromocytoma was established based on concordant imaging findings (abdominal CT and positron emission tomography revealing an adrenal mass) and biochemical confirmation with markedly elevated plasma and urinary metanephrine levels. This case highlights the importance of investigating secondary causes in recurrent or atypical TTS and emphasizes that, in the setting of PE associated with left ventricular dysfunction, concomitant cardiomyopathy should be considered rather than attributing findings solely to embolic severity.

## Introduction

Takotsubo syndrome (TTS) accounts for approximately 1-2% of patients presenting with suspected acute coronary syndrome and is characterized by transient left ventricular systolic dysfunction in the absence of obstructive coronary artery disease [1,2]. The pathophysiology is largely attributed to catecholamine excess, leading to myocardial stunning, coronary microvascular dysfunction, and direct myocardial toxicity [3]. Although most cases are idiopathic, up to 10-15% are secondary to identifiable triggers, particularly neurological or endocrine disorders [4]. Among these, pheochromocytoma represents a major but underrecognized cause, often associated with severe, recurrent, and atypical forms of TTS [5]. The association between TTS and pulmonary embolism (PE) is rare and may represent a diagnostic challenge. In such cases, left ventricular dysfunction may be misinterpreted as a marker of embolic severity, potentially delaying recognition of concomitant cardiomyopathy or underlying systemic disease [6,7]. We report a case of recurrent reverse Takotsubo cardiomyopathy associated with PE, ultimately revealing a pheochromocytoma.

## Case presentation

A 46-year-old woman with no prior cardiovascular history, no identified thromboembolic risk factors, and no chronic medication was admitted for recurrent episodes of acute chest pain. One year prior to the current hospitalization, she initially presented with acute chest pain associated with a moderate elevation of cardiac biomarkers, with troponin levels reaching 250 ng/L (normal: < 14 ng/L). In the context of suspected acute coronary syndrome, coronary angiography demonstrated normal coronary arteries, thereby excluding obstructive coronary artery disease (Figure 1). Left ventriculography revealed a pattern consistent with stress cardiomyopathy, with global left ventricular systolic dysfunction and an ejection fraction estimated at 39% in the right anterior oblique projection (Figure 2). Transthoracic echocardiography confirmed transient left ventricular systolic dysfunction. The clinical course was favorable, with complete recovery of left ventricular function on follow-up echocardiography.

**Figure 1:**
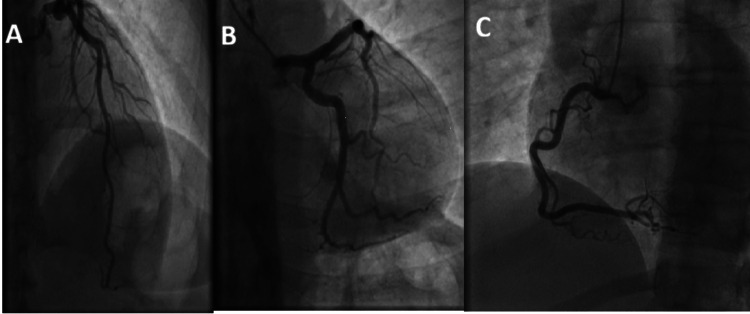
Coronary angiography demonstrating normal coronary arteries without evidence of obstructive disease (A) Left anterior descending artery, (B) left circumflex artery, and (C) right coronary artery.

**Figure 2:**
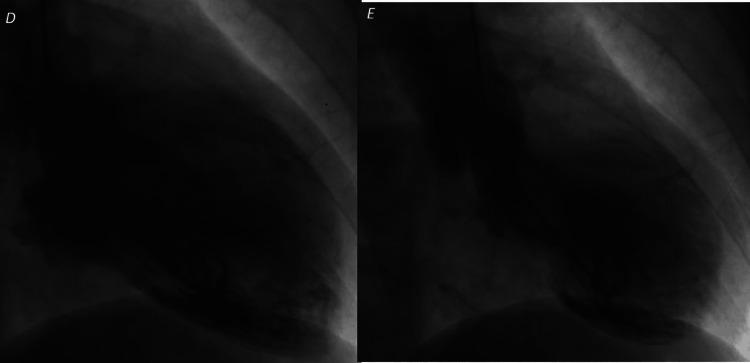
Left ventriculography demonstrated findings consistent with stress cardiomyopathy, with global left ventricular systolic dysfunction (ejection fraction of 39% in the right anterior oblique projection) and a characteristic apical ballooning pattern due to apical hypokinesia and basal hyperkinesia D: End diastole; E: End systole

One year later, the patient was readmitted for sudden-onset chest pain associated with severe dyspnea. On admission, clinical examination revealed cardiogenic shock with signs of low cardiac output, including hypotension, peripheral hypoperfusion, and signs of organ dysfunction. The patient required urgent hemodynamic support. No marked hypertensive episodes or paroxysmal blood pressure fluctuations suggestive of pheochromocytoma were observed at this stage. Laboratory investigations showed a marked elevation of cardiac biomarkers, with troponin levels reaching 1,679 ng/L (normal: < 14 ng/L). In this context of hemodynamic instability, an urgent transthoracic echocardiography was performed at the bedside in the intensive care unit, revealing severe left ventricular systolic dysfunction with an estimated ejection fraction of 10%, associated with global hypokinesia, more pronounced in the apical segments. The patient was admitted to the intensive care unit and managed with hemodynamic support, with progressive clinical improvement. A follow-up transthoracic echocardiography performed after discharge from the intensive care unit demonstrated complete recovery of left ventricular systolic function, confirming the reversible nature of myocardial dysfunction with an ejection fraction of 56% (Figure 3).

**Figure 3:**
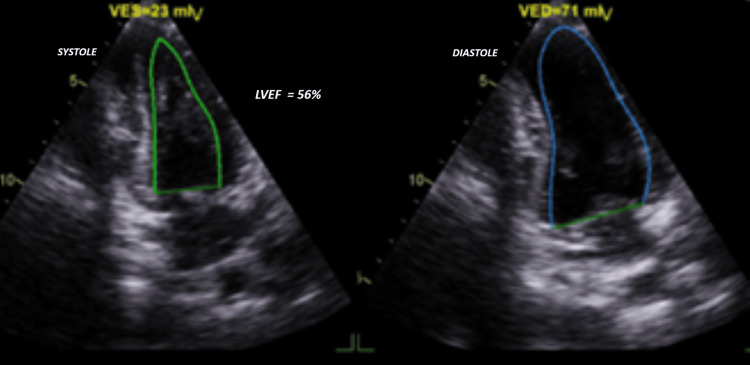
Follow-up transthoracic echocardiography demonstrated normalization of left ventricular systolic function, with a recovered ejection fraction of 56% LVEF: left ventricular ejection fraction

To further characterize myocardial involvement and exclude alternative diagnoses, cardiac magnetic resonance imaging was performed during the same hospitalization, following hemodynamic stabilization. The examination demonstrated a normal-sized left ventricle with complete recovery of systolic function (left ventricular ejection fraction (LVEF) estimated at 59%) and no regional wall motion abnormalities. Notably, no late gadolinium enhancement was observed, thereby excluding myocardial necrosis or fibrosis. This absence of late gadolinium enhancement represented a key diagnostic element supporting stress cardiomyopathy and effectively ruling out myocarditis. Right ventricular size and systolic function were also normal. Overall, these findings were consistent with a reversible myocardial injury compatible with TTS (Figure 4). Importantly, right ventricular size and function remained normal and non-dilated throughout both episodes.

**Figure 4:**
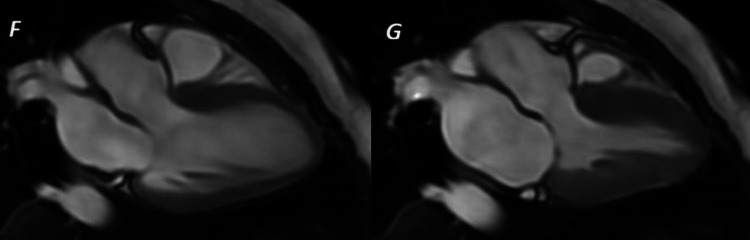
Cardiac magnetic resonance cine images in a four-chamber view (F) Image acquired during diastole showing a normal left ventricular cavity size with preserved myocardial thickness and right ventricular size and function. (G) Image acquired during systole demonstrating normal global left ventricular contraction without regional wall motion abnormalities.

Given the recurrence and severity of stress cardiomyopathy, an extensive etiological workup was undertaken to identify a potential underlying cause. A thoraco-abdominopelvic computed tomography scan revealed both a subsegmental PE and a right adrenal mass measuring 56 mm, highly suggestive of a functional adrenal tumor (Figure 5). Ventilation-perfusion scintigraphy confirmed multiple bilateral perfusion defects consistent with PE. The coexistence of recurrent stress cardiomyopathy, PE, and an adrenal mass raised a strong suspicion for an underlying systemic disorder. Subsequent positron emission tomography demonstrated intense focal uptake of the right adrenal lesion, with no evidence of extra-adrenal involvement, supporting the diagnosis of a functional adrenal tumor (Figure 6).

**Figure 5:**
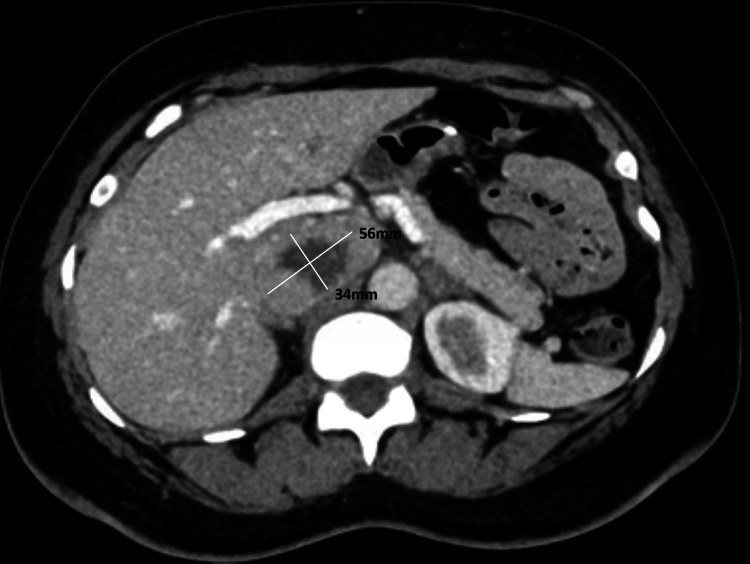
Contrast-enhanced thoraco-abdominopelvic CT demonstrated a well-defined right adrenal mass with imaging features suggestive of a functional adrenal tumor, consistent with a pheochromocytoma

**Figure 6:**
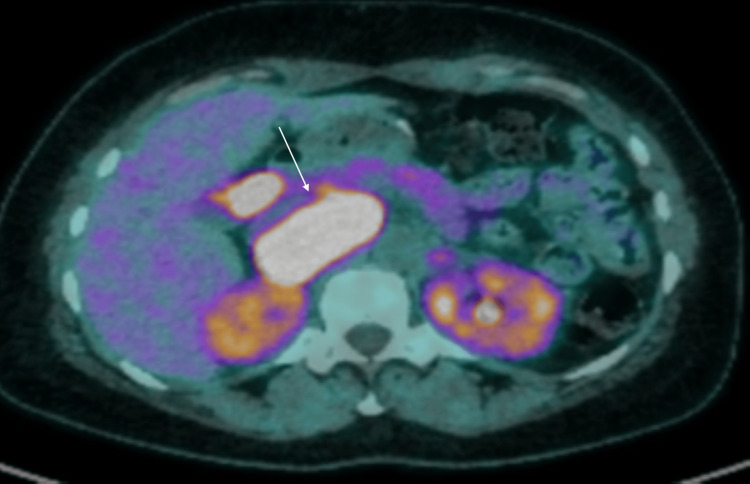
18F-DOPA PET imaging revealed a hypermetabolic right adrenal lesion with intense focal uptake, highly suggestive of a pheochromocytoma A single intensely hypermetabolic focus at the level of the right adrenal mass, consistent with the clinical suspicion of a pheochromocytoma.

Biochemical evaluation revealed markedly elevated catecholamine metabolites, with plasma metanephrines at 3.1 nmol/L (normal: < 0.5 nmol/L) and plasma normetanephrines at 4.0 nmol/L (normal: < 0.9 nmol/L). These values, exceeding several-fold the upper reference limits, were diagnostic of pheochromocytoma. The patient was subsequently referred to a specialized urological department and, following appropriate preoperative preparation with alpha-adrenergic blockade, underwent adrenalectomy.

## Discussion

The association between stress cardiomyopathy and PE remains rare and insufficiently described in the literature, particularly in case reports, such as that described by Jin et al. [6]. When reported, PE is most often considered a triggering factor for TTS through mechanisms involving acute physiological stress, hypoxemia, and intense neurohormonal activation [7]. However, this unidirectional relationship does not fully account for complex clinical presentations, particularly in cases of recurrence, severe hemodynamic compromise, or temporal dissociation between events. In such situations, the possibility of an underlying systemic condition should be carefully considered.

In our case, a particularly noteworthy feature was the variability in myocardial involvement across episodes. The first episode exhibited a typical pattern of TTS, characterized by apical hypokinesia and basal hyperkinesia. In contrast, the second episode, which was more severe and complicated by cardiogenic shock, presented with an atypical pattern, suggesting a different myocardial response in the setting of more intense catecholaminergic stress. This phenotypic heterogeneity is well described in secondary forms of stress cardiomyopathy and should raise suspicion for an underlying cause.

Among secondary etiologies, a pheochromocytoma represents a key but frequently underrecognized cause of stress cardiomyopathy. This rare neuroendocrine tumor, arising from chromaffin cells, is characterized by excessive and often paroxysmal catecholamine secretion, leading to a wide spectrum of cardiovascular manifestations [5,8]. These include acute cardiomyopathy, arrhythmias, hypertensive crises, and cardiogenic shock. Importantly, pheochromocytoma-associated cardiomyopathies tend to be more severe than idiopathic forms. Varghese et al. reported a higher incidence of cardiogenic shock and multiorgan failure in such patients [9]. The underlying pathophysiological mechanisms involve direct catecholamine-induced myocardial toxicity, diffuse coronary vasoconstriction, and microvascular dysfunction [3,10].

In this context, atypical forms of TTS have particular diagnostic value. While the classical apical ballooning pattern is the most common, several variants have been described, including basal, mid-ventricular, and focal forms. These atypical presentations are more frequently observed in secondary cardiomyopathies, particularly in the setting of pathological hypercatecholaminemia, such as a pheochromocytoma [11,12]. Therefore, the occurrence of a second, more severe, and atypical episode in our patient strongly supported the hypothesis of an underlying secondary cause and justified an extensive etiological workup.

Another important aspect highlighted by this case is the association between pheochromocytoma and thromboembolic complications. Although uncommon, venous thromboembolism has been reported with increased incidence in patients with a pheochromocytoma [13]. Several mechanisms may contribute to this prothrombotic state, including catecholamine-induced platelet activation, endothelial dysfunction, and alterations in coagulation pathways [13,14]. These mechanisms may explain the occurrence of PE in our patient, either independently or in combination with other transient risk factors.

From a diagnostic perspective, the coexistence of PE and elevated cardiac biomarkers represents a significant pitfall. In acute PE, troponin elevation is typically interpreted as a marker of right ventricular strain and is used for risk stratification [15]. However, severe left ventricular systolic dysfunction is not expected in non-massive PE. Therefore, the presence of marked left ventricular impairment, particularly when associated with characteristic wall motion abnormalities and subsequent recovery, should prompt consideration of concomitant stress cardiomyopathy. Failure to recognize this association may lead to overestimation of embolic severity and delay the diagnosis of an underlying condition such as a pheochromocytoma.

Overall, this case illustrates a complex interplay between stress cardiomyopathy, PE, and catecholamine excess due to pheochromocytoma. The recurrence, severity, and especially the transition from a typical to an atypical presentation were key elements that prompted further investigations and ultimately led to the diagnosis of pheochromocytoma.

## Conclusions

The rare association of recurrent stress cardiomyopathy and PE led, in this case, to the diagnosis of a previously unrecognized pheochromocytoma. This case highlights that recurrent and severe stress cardiomyopathy should prompt investigation for an underlying cause, particularly a pheochromocytoma. It also emphasizes that left ventricular dysfunction in the setting of PE, especially when associated with unexplained ventricular dysfunction and elevated cardiac biomarkers, particularly troponin levels, should not be automatically attributed to embolic severity, but may reflect concomitant stress cardiomyopathy.
